# Low levels of tumour suppressor miR-655 in plasma contribute to lymphatic progression and poor outcomes in oesophageal squamous cell carcinoma

**DOI:** 10.1186/s12943-018-0929-3

**Published:** 2019-01-04

**Authors:** Jun Kiuchi, Shuhei Komatsu, Taisuke Imamura, Keiji Nishibeppu, Katsutoshi Shoda, Tomohiro Arita, Toshiyuki Kosuga, Hirotaka Konishi, Atsushi Shiozaki, Kazuma Okamoto, Hitoshi Fujiwara, Daisuke Ichikawa, Eigo Otsuji

**Affiliations:** 1 Division of Digestive Surgery, Department of Surgery, Kyoto Prefectural University of Medicine, 465 Kajii-cho, Kawaramachihirokoji, Kamigyo-ku, Kyoto, 602-8566 Japan; 2 First Department of Surgery, Faculty of Medicine, University of Yamanashi, Yamanashi, Japan

**Keywords:** Plasma microRNA, Oesophageal squamous cell carcinoma, Mouse model, Lymph node metastasis, Biomarker, Therapeutic agent

## Abstract

Recent studies identified that low levels of tumour suppressor microRNAs (miRNAs) in plasma/serum relate to tumour progression and poor outcomes in cancers. We selected six candidates (miR-126, 133b, 143, 203, 338-3p, 655) of tumour suppressor miRNAs in oesophageal squamous cell carcinoma (ESCC) by a systematic review of NCBI database. Of these, miR-655 levels were significantly down-regulated in plasma of ESCC patients compared to healthy volunteers by test- and validation-scale analyses. Low levels of plasma miR-655 were significantly associated with lymphatic invasion, lymph node metastasis and advanced stage. Univariate and multivariate analysis revealed that the low level of plasma miR-655 was an independent risk factor of lymphatic progression and a poor prognostic factor. Overexpression of miR-655 in ESCC cells inhibited cell proliferation, migration, invasion and epithelial-mesenchymal transition. Increased plasma miR-655 levels by the subcutaneous injection significantly inhibited lymph node metastasis in mice. Low levels of miR-655 in plasma relate to lymphatic progression and poor outcomes, and the restoration of the plasma miR-655 levels might inhibit tumour and lymphatic progression in ESCC.

**Electronic supplementary material:**

The online version of this article (10.1186/s12943-018-0929-3) contains supplementary material, which is available to authorized users.

Numerous studies have attempted to understand the molecular mechanisms of tumorigenesis and identify clinical biomarkers and molecular targets for oesophageal squamous cell carcinoma (ESCC). Regarding microRNAs (miRNAs), many studies have identified alterations in miRNA expressions that are correlated with the progression of various diseases including the development and progression of cancers [1, 2]. Furthermore, in the last decade, numerous blood-based miRNAs including our studies have been detected as biomarkers for cancer detection, monitoring tumour dynamics and predicting prognosis and chemoresistance [3–7].

Recently, Kosaka and Ochiya et al. suggested a novel theory from their in vitro study [8]. Namely, miRNAs facilitate the system of maintenance and surveillance against cancer progression; tumour suppressor miRNAs are normally secreted from neighboring healthy cells to cancer cells to inhibit tumour progression. Delivered tumour suppressor miRNAs are taken up by cancer cells and might function as an anti-tumour molecule. During the early stage of tumourigenesis, the depletion of tumour suppressor miRNAs by cancer cells may be compensated for by the surrounding healthy cells, which supply exosomes containing tumour suppressor miRNAs. However, once the surrounding cells can no longer meet this demand, cancer cells progress to an advanced stage [8].

Because blood-based miRNAs are considered to be released from cancer tissues as well as normal tissues, most of these miRNAs are expected to have originated from normal tissues; thus, we hypothesized that tumour suppressor miRNAs might become depleted from healthy cells in accordance with cancer progression. Consequently, we demonstrated in clinical settings that the down-regulated tumour suppressor miRNAs in plasma were associated with worse survival [9, 10].

In this study, we focused on tumour suppressor miRNAs that are down-regulated in the plasma of ESCC patients, and we investigated the usefulness of these tumour suppressor miRNAs as biomarkers and therapeutic agents for ESCC. Materials and methods are shown in additional files (Additional file 1: Supplementary materials and methods, Additional file 2: Figure S4, Additional file 3: Table S6 and Additional file 4: Table S7).

## Results and discussion

### Selection of plasma miRNA candidates based on a systematic review of the NCBI database

This study was designed as follows: (1) selection of candidate miRNAs based on a systematic review of the NCBI database; (2) test-scale analysis of plasma samples using qRT-PCR to validate the utility of the selected miRNA candidates; (3) validation-scale analysis of the miR-655 plasma level to investigate the correlations of the miR-655 plasma level with clinicopathological characteristics and prognostic outcomes in ESCC patients; (4) evaluation of whether miR-655 overexpression in ESCC cells induced anti-tumour effects in vitro; and (5) investigation of the tumour suppressor function in tumor and lymph node metastasis in vivo (Additional file 5: Figure S1a and S1b).

We searched for all studies related to ESCC miRNAs in PubMed up to June 2016 and selected 58 candidate miRNAs as shown in Additional file 5: Figure S1a, Additional file 6: Table S1 and Additional file 1: Supplementary Materials and Methods. After a series of exclusion criteria were applied, we consequently selected six candidate miRNAs: miR-126 [11], miR-133b [12], miR-143 [13], miR-203 [14], miR-338-3p [15], and miR-655 [16].

### Test-scale analysis of the plasma levels of six miRNAs in ESCC patients and healthy volunteers

First, we investigated the plasma levels of the six selected miRNAs in 10 ESCC patients and 10 healthy volunteers by qRT-PCR using a test-scale analysis. As shown in Additional file 5: Figure S1c, the levels of plasma miR-143 and miR-655 were significantly down-regulated in ESCC patients compared with healthy volunteers. To assess the therapeutic potential of tumor suppressor miRNAs, we planned an animal experiment in which tumor suppressor miRNA was administered to mice and the tumor suppressive function of the given miRNAs was assessed. In the experiment, miRNAs that do not originally exist in mice were considered to be suitable for assessing the dynamics of administered miRNA. The presence of miR-143 has previously been reported in mice organs, but that of miR-655 has not been reported in mice organs [17–19]. Thus, we finally selected miR-655 for further analysis in this study.

### Validation-scale analysis of the miR-655 plasma levels in ESCC patients

**Fig. 1:**
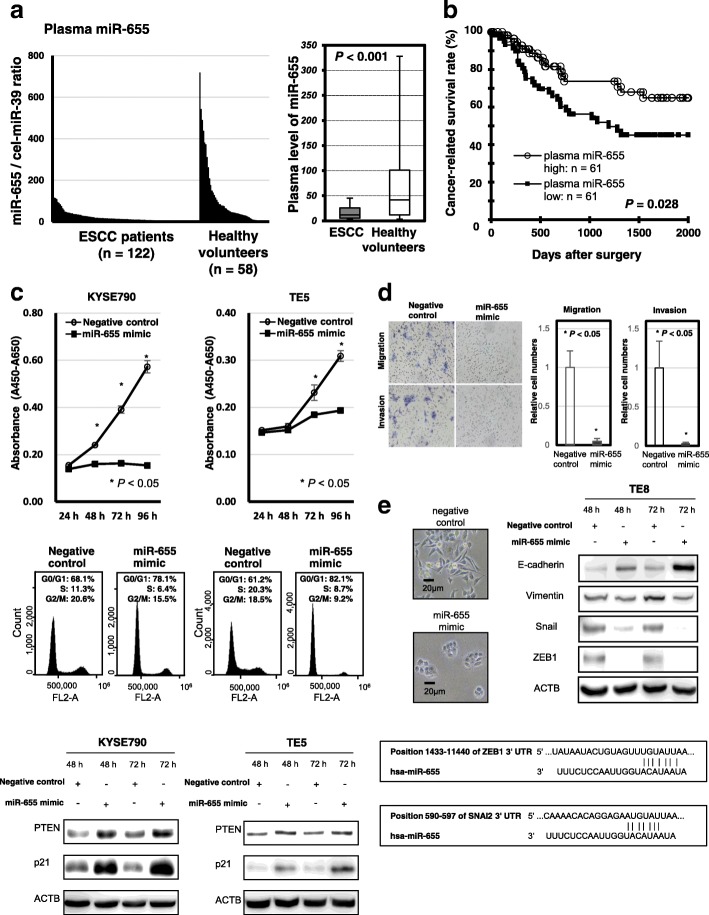
Large-scale analysis of the miR-655 plasma levels in ESCC patients and healthy volunteers, and investigation whether miR-655 would suppress tumour progression and EMT in ESCC cells. **a** We confirmed that the plasma levels of miR-655 was significantly lower in ESCC patients than in healthy volunteers (*P* < 0.001). **b** Decreased plasma miR-655 was associated with poor prognostic outcomes in ESCC patients (*P* = 0.028). **c** Cell proliferation analysis by miR-655 overexpression. Proliferation was significantly suppressed in KYSE790 and TE5 cells transfected with the miR-655 mimic compared with cells transfected with the negative control mimic. The FACS analysis demonstrated that transfection of KYSE790 and TE5 cells with the miR-655 mimic resulted in an accumulation of cells in the G1-S phase compared with transfection with the control miRNA mimic. In the protein analysis, overexpression of miR-655 induced the production of p21 and PTEN proteins at 72 h after transfecting the cells with the miR-655 mimic. **d** Trans-well migration and invasion assays demonstrated that miR-655 suppressed the ability of ESCC cells to migrate and invade. **e** Overexpression of miR-655 induced a significant morphological change, increased the expression of E-cadherin protein, and reduced the expression of Vimentin, Snail, and ZEB1 proteins

Next, we validated our observations in a large-scale setting. We observed that the miR-655 plasma levels were significantly lower in the ESCC patients than in the healthy volunteers (*p* < 0.001; Fig. 1a). We utilized the AUC value and the Youden index and found that the AUC value was 0.782 [20]. The optimal relative cut-off point was indicated to be 26.57, with a sensitivity of 79.5% and a specificity of 67.3%. Our results provide evidence that the miR-655 plasma level can be used to distinguish ESCC patients from healthy volunteers to a clinically satisfactory degree in comparison with conventional tumor markers. (Additional file 7: Figure S2a).

### miR-655 levels in normal organs and ESCC cell lines

As shown in Additional file 7: Figure S2b, miR-655 was highly expressed in the brain, testis, colon, and oesophagus. In all ESCC cell lines, the miR-655 levels were lower than in the normal oesophageal mucosa.

### Investigation of whether plasma miR-655 levels were associated with miR-655 expressions in tissues and exosomes

The expression levels of miR-655 were significantly higher in non-cancerous tissues than in cancerous oesophageal tissues (*P* < 0.01), and the plasma levels of exosomal miR-655 were significantly down-regulated in ESCC patients than in healthy volunteers (*P* < 0.05) (Additional file 7: Figure S2c and S2d).

### Potential utility of plasma miR-655 as a prognostic biomarker in ESCC patients

Prognostic analysis revealed that low miR-655 plasma levels were significantly associated with a worse cancer-related survival rate in ESCC patients (*P* = 0.028) (Fig. 1b). Furthermore, a low miR-655 plasma level was an independent factor predicting poor prognosis in ESCC patients (*P* = 0.021; hazards ratio, 2.34; 95% confidence interval [CI]: 1.11–5.92) (Additional file 8: Table S2).

### Correlation between plasma miR-655 levels and clinicopathological characteristics in ESCC patients

We analyzed the correlation between plasma miR-655 levels and clinicopathological characteristics in 122 ESCC patients (Table 1). A low miR-655 plasma levels were significantly correlated with older age (*P* = 0.019), advanced N stage (*P* = 0.025), advanced lymphatic invasion (*P* = 0.004), and advanced pathological stage (*P* = 0.041). To evaluate the effect of low level of plasma miR-655 on tumor aggressiveness in each stage, we analyzed the association between plasma miR-655 levels and clinicopathological factors in early Stage I and advance Stage II-III (Additional file 9: Table S3 and Additional file 10: Table S4). A low miR-655 plasma levels were significantly associated with advanced lymphatic invasion in each stage (Stage I: *P* = 0.021, Stage II-III: *P* = 0.023) and positive lymph node metastasis in Stage II-III (*P* = 0.016).

**Table 1:** Association between plasma miR-655 levels and clinicopathological characteristics in ESCC patients

		Plasma miR-655 concentration				
Variables		High (*n* = 61)		Low (*n* = 61)		*P*-value^a^
Gender	Female	14	(61%)	9	(39%)	0.354
	Male	47	(47%)	52	(53%)	
Age (60 years old)	< 60	17	(74%)	6	(26%)	**0.019**
	60 <	44	(44%)	55	(56%)	
T factor	T1,2	31	(50%)	31	(50%)	1.000
	T3,4	30	(50%)	30	(50%)	
N factor	N0,1	50	(56%)	39	(44%)	**0.025**
	N2,3,4	11	(33%)	22	(67%)	
Lymphatic invasion	ly0,1	56	(63%)	33	(37%)	**0.004**
	ly2,3	5	(15%)	28	(85%)	
Venous invasion	v0,1	48	(51%)	46	(49%)	0.243
	v2,3	8	(36%)	14	(64%)	
p Stage	Stage I	13	(72%)	5	(28%)	**0.041**
	Stage II, III, IV	48	(46%)	56	(54%)	
Tumor size	< 50 mm	36	(54%)	31	(46%)	0.466
	50 mm <	25	(45%)	30	(55%)	
Histology	Well and moderately differentiated	44	(48%)	47	(52%)	0.677
	Poorly differentiated	17	(55%)	14	(45%)	
Recurrences	Absent	39	(57%)	30	(43%)	0.143
	Present	22	(42%)	31	(58%)	

^a^ Chi-square or Fisher tests. NOTE: significant values are in bold

Next, we compared the clinicopathological characteristics between patients with and without active lymphatic progression. Multivariate analysis revealed that a low plasma level of miR-655 was an independent risk factor for active lymphatic progression (*P* = 0.001, Odds ratio 3.25) (Additional file 11: Table S5). In these analyses, we found that low plasma levels of miR-655 were associated with lymph node metastasis, which is known as one of the most important factors for poor prognosis in ESCC [21]. Therefore, we planned in vitro and vivo analyses to investigate whether treatment with miR-655 could suppress tumour progression, especially lymph node metastasis.

### Investigation of the tumour suppressive function of miR-655 in ESCC cells

We confirmed the tumour suppressive function of miR-655 in ESCC cells by the cell proliferation assay, the FACS analysis, the trans-well migration and invasion assays and the protein analysis using miRNA mimics (KYSE790 and TE5 in Fig. 1c and d; the other cell lines in Additional file 12: Figure S3). Moreover, we confirmed that miR-655 could suppress EMT in ESCC cells as previously reported [16] (Fig. 1e).

### Accumulation and maintenance of the tumour suppressor miR-655 in plasma suppressed lymph node metastasis without side effects in vivo

**Fig. 2:**
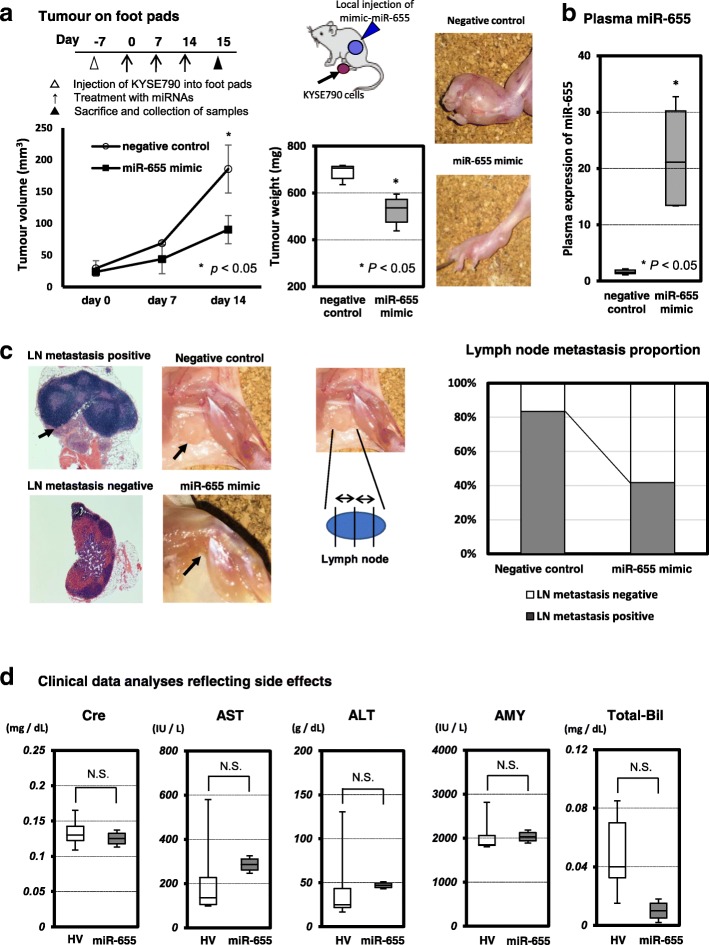
Restoration of the miR-655 plasma levels could suppress tumour growth and lymph node metastasis in vivo. **a** Investigation into whether miR-655 could suppress tumour growth in vivo. The popliteal lymph node metastasis model was used to assess the therapeutic effect of miR-655 for lymph node metastasis. Seven days after the injection of KYSE790 cells into the footpads, tumour development at the injection site was visually confirmed every 7 days. The miR-655 or negative control mimic with atelocollagen was subcutaneously injected into the ventral surface of the lower flank region, which is far from the region injected with cancer cells, and these treatments continued weekly for three weeks after the initial treatment. The treatment with miR-655 mimic significantly suppressed tumour growth on the footpads compared with the negative control mimic. **b** Comparison of the plasma miR-655 levels between mice treated with miR-655 mimic and treated with negative control mimic. The plasma levels of miR-655 were significantly higher in mice treated with miR-655 mimic than in mice treated with negative control mimic. **c** Investigation into whether treatment with miR-655 mimic could suppress lymph node metastasis in vivo. To diagnose the lymph node metastasis, we resected the popliteal lymph node on the affected side and sectioned each lymph node into three slices. Lymph node metastasis was diagnosed by hematoxylin–eosin staining. The proportion of lymph node metastasis was significantly lower in mice treated with miR-655 mimic than in mice treated with negative control mimic. **d** Blood data analyses reflecting potential side effects following miR-655 treatment. There were no side effects in blood-based parameters of organ disorders such as creatinine (Cre), aspartate aminotransferase (AST), alanine aminotransferase (ALT), amylase (AMY), and total bilirubin (T-Bil)

To examine the possible tumour suppressive function of miR-655 in vivo, we used the popliteal lymph node metastasis xenograft model [22]. As a result, the miR-655 mimic more significantly suppressed tumour growth on the footpads than the negative control mimic (Fig. 2a). Regarding the plasma levels of miR-655, miR-655 levels were significantly higher in mice treated with miR-655 mimic than in mice treated with the negative control mimic (Fig. 2b).

As shown in Fig. 2c, the proportion of lymph node metastasis was significantly lower in mice treated with miR-655 mimic than in mice treated with negative control mimic. These results strongly suggested that the treatment with miR-655 could suppress lymph node metastasis.

Concerning the molecular functions of miR-655, various functions have been reported in cancers such as lung adenocarcinoma [23], ovarian cancer [24], acute myeloblastic leukemia [25], breast cancer [26], hepatocellular carcinoma [27], colorectal cancer [28] and ESCC [16]. Specifically, miR-655 targets various oncogenes and inhibits their functions by targeting Prrx1 in breast cancer [26], ADAM10 in hepatocellular carcinoma, and ZEB1, TGFBR2, PTTG1 in ESCC [16, 29]. Prrx1, ZEB1, and TGFR2 are known as key molecules in EMT progression. These previous studies support our data about the tumor suppressor function of miR-655, especially for inhibiting tumor and lymphatic progression in thorough cell cycle arrest and MET in vitro in ESCC.

Recently, two promising clinical studies regarding the clinical application of miRNAs have been performed. The first study focused on the therapeutic silencing of disease-associated miRNAs using miRNA inhibitors. Miravirsen (Santaris Pharma) is one of several promising miRNA inhibitors; it can bind to miR-122 and inhibit its biogenesis. Miravirsen was developed for the treatment for hepatitis C and is currently under evaluation in clinical trials [30, 31]. The second study focused on therapeutic miRNA-based drugs using synthetic miRNA mimics. MIRX34 (Mirna Therapeutics, Inc.) is a synthetic miRNA mimic of the tumour suppressor miR-34, and a phase I clinical trial using MIRX34 for patients with primary or metastatic liver cancer was performed [32]. Whereas this trial was ended because of serious adverse immune-related effects, administration of miR-655 in mice did not cause any clinical adverse events or side effects in blood-based parameters reflecting organ disorders in our study (Fig. 2d). Moreover, plasma miR-655 is depleted in ESCC patients. Therefore, we believe that the restoration of tumour suppressor miR-655, which is abundantly detected in the plasma of healthy human individuals, may be a safe treatment for minimizing various physiological risks in clinical applications. Indeed, we have already reported that the restoration of depleted tumor suppressor plasma miR-107 to the same level as healthy individuals suppressed tumor progression and caused no significant adverse events in mice bearing pancreatic cancer cells [10].

Because plasma miR-655 level was significantly down-regulated in ESCC patients compared with healthy volunteers, the restoration of plasma miR-655 level might be a novel and safe anticancer therapy for inhibiting tumor progression and lymph node metastasis in ESCC patients. However, many issues must still be addressed before these findings can be translated into a clinically useful treatment agent for ESCC patients. Although we confirmed that miR-655 was incorporated into exosomes and that exosomal miR-655 was down-regulated in the plasma of the ESCC patients compared with that of healthy volunteers (Additional file 7: Figure S2d), we could not technically use exosomal miR-655 but instead subcutaneously injected mimic miR-655 with atelocollagen. In clinical settings, subcutaneous injection of mimic miR-655 with atelocollagen may be a safe and cost-effective method. However, further studies are needed on the effective control of cellular uptake and the secretion systems of tumor suppressor miRNAs, leading to the development of miRNA delivery systems for future therapeutic and diagnostic applications [33, 34]. These studies are currently under evaluation.

## Conclusions

We demonstrated that low levels of miR-655 in plasma were significantly related to lymphatic progression and poor outcomes in ESCC. Overexpression of miR-655 in ESCC cells suppressed tumor progression. In the in vivo analyses, increased plasma miR-655 levels significantly inhibited the lymphatic progression. These results suggested that plasma miR-655 levels could play pivotal roles in lymphatic progression in ESCC patients. MiR-655 is warranted for further investigation as a novel therapeutic agent.

## Additional files

Additional file 1: Supplementary materials and methods. (DOCX 48 kb)

Additional file 2: **Figure S4.** Identification of the appropriate ESCC cell lines for investigation into whether miR-655 can suppress EMT. The panel mRNA expression levels of E-cadherin and Vimentin were investigated, and the Vimentin / E-cadherin ratio was calculated. In TE8 cells, Vimentin mRNA expression was high and E-cadherin mRNA expression was low. (PDF 85 kb)

Additional file 3: **Table S6.** Clinical data of ESCC patients. (DOCX 47 kb)

Additional file 4: **Table S7.** The primer sequences used in this study. (DOCX 16 kb)

Additional file 5: **Figure S1.** Study design and selection of plasma miRNA candidates. (a) A systematic review of the NCBI database to identify novel plasma biomarkers of miRNA in patients with ESCC. This study was designed as follows: (1) selection of candidate miRNAs based on a systematic review of the NCBI database; (2) test-scale analysis of plasma samples using qRT-PCR to validate the utility of the selected miRNA candidates; (3) validation-scale analysis of the miR-655 plasma levels to investigate the correlations of the miR-655 plasma levels with clinicopathological characteristics and prognostic outcomes in ESCC patients; (4) evaluation of whether miR-655 overexpression in ESCC cells induced anti-tumour effects in vitro; and (5) investigation of the tumour suppressive function in tumour and lymph node metastasis in vivo. (b) Study design to find novel candidate miRNAs that decrease in patient plasma as a therapeutic target for ESCC. After a series of exclusion criteria were applied, we consequently selected six candidate miRNAs: miR-126, 133b, 143, 203, 338-3p, and 655. (c) Small-scale analysis of the plasma levels of six miRNAs in ESCC patients and healthy volunteers by qRT-PCR. We investigated the plasma levels of the six selected miRNAs in 10 ESCC patients and 10 healthy volunteers by qRT-PCR. The plasma levels of miR-143 and miR-655 were significantly down-regulated in ESCC patients compared to healthy volunteers, and we selected miR-655 for further analysis. (PDF 30 kb)

Additional file 6: **Table S1.** Selected process of all candidate miRnas. (PDF 55 kb)

Additional file 7: **Figure S2.** MiR-655 levels in plasma, normal human organs and cell lines. (a) Receiver-operating characteristic (ROC) curves and area under the ROC curve (AUC) values were used to assess the feasibility of using plasma miRNA levels as a diagnostic tool for detecting ESCC (AUC 0.782). The optimal relative expression cut-off point was found to be 26.57 using the miR-655 / cel-miR-39 ratio, with a sensitivity of 79.5% and a specificity of 67.3%. (b) miR-655 levels in normal human organs and ESCC cell lines. miR-655 was highly expressed in the brain, testis, colon, and oesophagus. In all ESCC cell lines, the expression of miR-655 was lower than in the normal oesophageal mucosa. (c) miR-655 levels in non-cancerous and ESCC tissues. The expression of miR-655 was significantly higher in the non-cancerous oesophageal mucosa than in ESCC tissues (*P* < 0.01). (d) miR-655 levels in exosomes extracted from the plasma of ESCC patients and healthy volunteers. The expression levels of miR-655 in exosomes extracted from plasma was investigated in 7 ESCC patients and 7 healthy volunteers. Exosomal miR-655 in plasma was significantly down-regulated in ESCC patients compared to healthy volunteers (*P* < 0.05). (PDF 89 kb)

Additional file 8: **Table S2.** Univariate and multivariate analysis of ESCC patient survival following esophagectomy using the Cox proportional hazards model. (DOCX 17 kb)

Additional file 9: **Table S3.** Association between plasma miR-655 levels and clinicopathological characteristics in ESCC patients who were pathologically classified into Stage I. (DOCX 19 kb)

Additional file 10: **Table S4.** Association between plasma miR-655 levels and clinicopathological characteristics in ESCC patients who were pathologically classified into Stage II and III. (DOCX 20 kb)

Additional file 11: **Table S5.** Association between lymphatic progression and clinicopathological characteristics in ESCC patients. (DOCX 19 kb)

Additional file 12: **Figure S3.** Investigation into the tumour suppressive function of miR-655 in ESCC cells. Overexpression of miR-655 by miR-655 mimic induced the expression of p21 and PTEN proteins and significantly suppressed cell proliferation in TE2, TE8, and KYSE150 cells. (PDF 79 kb)

## Abbreviations

ALT: Alanine aminotransferase
AMY: Amylase
ARRIVE: Animal Research: Reporting of In Vivo Experiments
AST: Aspartate aminotransferase
AUC: Area under the ROC curve
CI: Confidence interval
Cre: Creatinine
EMT: Epithelial-mesenchymal transition
ESCC: Oesophageal squamous cell carcinoma
HDL: High density lipoprotein
HR: Hazard ratio
LN: Lymph node
MET: Mesenchymal endothelial transition
miRNA: MicroRNA
NCBI: The National Center for Biotechnology Information
qRT-PCR: Quantitative real-time polymerase chain reaction
RNU6B: U6 small nuclear RNA
ROC: Receiver-operating characteristic
T-Bil: Total bilirubin
