# Supplementary material for: Low levels of tumour suppressor miR-655 in plasma contribute to lymphatic progression and poor outcomes in oesophageal squamous cell carcinoma
Source: Mol Cancer. 2019 Jan 4;18:2. doi: 10.1186/s12943-018-0929-3 (PMC6320607; doi:10.1186/s12943-018-0929-3)
Supplement: Supplementary file 1 — Supplementary materials and methods. (DOCX 48 kb) [file 12943_2018_929_MOESM1_ESM.docx]

**Additional file 1: Supplementary materials and methods**

***Patients and samples***

All experimental methods were carried out in accordance with relevant guidelines and regulations. Written informed consent was obtained from all patients to use their tissue specimens and blood samples. This study was approved by the institutional review boards of Kyoto Prefectural University of Medicine (ERB-C-319-1). Between June 2010 and May 2015, a total of 122 plasma samples from consecutive ESCC patients and 52 plasma samples from healthy volunteers were collected. Patients with past caner history were excluded. Clinical data of ESCC patients are available in **Supplementary Table S6**. All tissue and plasma samples were obtained from Kyoto Prefectural University of Medicine. The 52 plasma samples from healthy volunteers included those from medical personnel and patients with benign disease, such as cholecystolithiasis and inguinal hernia. The healthy volunteers underwent medical examinations, such as blood analysis, endoscopy and computed tomography, and they were not shown to have any esophageal or cancerous diseases. Tumor stages were assessed according to the Union for International Cancer Control classification system [1].

A total of 7 mL peripheral blood was obtained from each patient before surgery and from the healthy volunteers. The blood was transferred into sodium heparin tubes (BD Vacutainer, Franklin Lakes, NJ) and immediately subjected to the three-spin protocol (1,500 r.p.m. for 30 min, 3,000 r.p.m. for 5 min, and 4,500 r.p.m. for 5 min) to prevent contamination by cellular nucleic acids. Then, the obtained plasma was stored at -80 °C until further processing. The resected specimens were fixed in formalin and embedded in paraffin for pathological diagnosis. Histological evaluation was performed for tissues adjacent to specimens according to the criteria of the World Health Organization. In all cases, at least two pathologists agreed with the pathological observations and confirmed the diagnosis.

***RNA extraction***

Total RNA was extracted from 400 μL of plasma using a mirVana PARIS Kit (Ambion, Austin, TX) and finally eluted into 100 μL of preheated (95 °C) elution solution according to the manufacturer’s protocol. A volume of 400 μL of plasma was used as the common denominator because there was no definite internal control in the plasma miRNA analyses, as shown in our previous studies [2] [3] [4] [5] [6] [7] [8]. Total RNA was also extracted from four 15-μm-thick slices of formalin-fixed and paraffin-embedded tissue samples (60-μm thickness) using a Recover All Total Nucleic Acid Isolation Kit (Ambion, Austin, TX) and subsequently eluted into 60 μL of Elution Solution according to the manufacturer’s protocol.

***A systematic review of the NCBI database to select candidate miRNAs***

We performed a systematic review of the NCBI database to identify novel plasma biomarkers of miRNA in patients with ESCC (**Supplementary Figure S1a** and **Supplementary Table S1**). We searched for all studies related to ESCC miRNAs in PubMed published before June 2016. This search was based on the key terms “Esophageal squamous cell carcinoma” and “tumor-suppressor microRNA”, and miRNAs reported to be down-regulated in ESCC tissue were selected as candidates. Any meeting abstracts not accompanied by full articles and other incomplete and non-English articles were excluded. The candidate miRNAs had to meet the following criteria: I) Candidate miRNAs reported several times with sufficient data in ESCC; II) Candidate miRNAs not previously reported as biomarkers in body fluids, such as plasma, serum, urine and semen. Two authors independently reviewed all articles identified in this search using these criteria, and a third author resolved any discrepancies.

***Quantification of miRNA by qRT-PCR***

The amounts of miRNAs were quantified by qRT-PCR using a Human TaqMan MicroRNA Assay Kit (Applied Biosystems, Foster City, CA). The reverse transcription reaction was conducted with a TaqMan MicroRNA Reverse Transcription Kit (Applied Biosystems) in 5 μL of solution containing 1.67 μL of extracted RNA, 0.05 μL of 100 mM dNTPs, 0.33 μL of Multiscribe Reverse Transcriptase (50 UμL^-1^), 0.5 μL of 10 × reverse transcription buffer, 0.06 μL of RNase inhibitor (20 UμL^-1^), 1 μL of gene-specific primer (hsa-miR-126, Assay ID: 002228; hsa-miR-133b, Assay ID: 002247; hsa-miR-143, Assay ID: 002249; hsa-miR-203, Assay ID: 000507; hsa-miR-338-3p, Assay ID: 002252; hsa-miR-655, Assay ID: 001612), and 1.39 μL of nuclease-free water. The primer sequences used in this study are reported in **Supplementary Table S7**. To synthesize cDNA, reaction mixtures were incubated at 16 °C for 30 min, at 42 °C for 30 min, and at 85 °C for 5 min, and were then held at 4 °C. Next, 0.67 μL of cDNA was amplified using 5 μL of TaqMan 2 × Universal PCR Master Mix with no AmpErase UNG (Applied Biosystems), 0.5 μL of gene-specific primers/probes and 3.83 μL of nuclease-free water in a final volume of 10 μL. qPCR was run on a StepOnePlus PCR system (Applied Biosystems), and reaction mixtures were incubated at 95 °C for 10 min, followed by 40 cycles of 95 °C for 15 s and 60 °C for 1 min. Cycle threshold (Ct) values were calculated with StepOne Software v2.0 (Applied Biosystems).

As shown in previous reports, we used an approach for data normalization based on spiking the samples with a synthetic RNA oligonucleotide, cel-miR-39, which does not exist in the human genome [9]. *C. elegans* cel-miR-39 was purchased as a custom-made RNA oligonucleotide (Qiagen, Valencia, CA). The oligo used for spiking, as a mixture of 25 fmol of oligonucleotide in a total water volume of 5 μL, was introduced after the addition of 2 × denaturing solution (Ambion) to the plasma sample to avoid degradation by endogenous plasma RNases. As a control for each RNA sample, cel-miR-39 was used for the TaqMan qRT-PCR assays (Applied Biosystems) as described above. We normalized the data across samples using the 2^－ΔΔCt^ method relative to celmiR-39. However, the expression of miRNAs from human tissue samples and cultured cells was normalized using the 2^－ΔΔCt^ method relative to U6 small nuclear RNA (RNU6B). ΔCt was calculated by subtracting the Ct values of cel-miR-39 or RNU6B from those of the miRNAs of interest. ΔΔCt was then calculated by subtracting the mean of ΔCt of healthy volunteer plasma or normal esophageal tissue from the ΔCt of ESCC plasma or tissues. The change in gene expression was calculated using the 2^－ΔΔCt^ method [10, 11].

***Quantification of mRNA by qRT-PCR***

Single-stranded complementary DNA generated from total RNA was ampliﬁed with primers speciﬁc for each gene, such as E-cadherin and Vimentin. The amounts of mRNAs were quantified by qRT-PCR using a Human TaqMan Gene Expression Assays (Applied Biosystems). The reverse transcription reaction was conducted with a TaqMan MicroRNA Reverse Transcription Kit (Applied Biosystems) in 20 μL of solution containing 10 μL of extracted RNA, 0.8 μL of 100 mM dNTPs, 1.0 μL of Multiscribe Reverse Transcriptase (50 UμL^-1^), 2.0 μL of 10 × Reverse Transcription Buffer, 1.0 μL of RNase inhibitor (20 UμL^-1^), 2.0 μL of 10 × RT random primer and 3.2 μL of nuclease-free water. To synthesize cDNA, reaction mixtures were incubated at 25 °C for 10 min, at 37 °C for 120 min, and at 85 °C for 5 min, and were then held at 4 °C. Next, 1.4 μL of cDNA was amplified using 10 μL of TaqMan 2 × Universal PCR Master Mix with no AmpErase UNG (Applied Biosystems), 1.0 μL of gene-specific primers/probes (Hs00170423_m1 for E-cadherin and Hs00185584_m1 for Vimentin; Applied Biosystems) and 7.6 μL of nuclease-free water in a final volume of 20 μL. qPCR was run on a StepOnePlus PCR system (Applied Biosystems), and reaction mixtures were incubated at 95 °C for 10 min, followed by 40 cycles of 95 °C for 15 s and 60 °C for 1 min. Ct values were calculated with StepOne Software v2.0 (Applied Biosystems).

***Culture of ESCC cell lines***

ESCC cell lines such as TE2 (CVCL 4455), TE5 (CVCL 1764), TE8 (CVCL 1766), TE9 (CVCL 1767), TE15 (CVCL 1763), KYSE150 (CVCL 1348), KYSE170 (CVCL 1358), and KYSE790 (CVCL 8510) were purchased from RIKEN Cell Bank (Tsukuba, Japan) and cultured in Roswell Park Memorial Institute 1640 medium (Sigma, St. Louis, MO) supplemented with 10% fetal bovine serum (Trace Scientific, Melbourne, Australia). All cells were cultured in 5% carbon dioxide at 37 °C in a humidified chamber. All cell lines were identified by Short Tandem Repeat profiling by the RIKEN Cell Bank (Tsukuba, Japan). No mycoplasma contamination was detected in any of the cultures.

***Transfection of ESCC cells with miRNA mimics***

For the overexpression of miR-655, the miR-655 mimic (Assay ID: MC11619) or negative control mimic miRNA (mirVana miRNA mimic Negative Control #1), both of which were selected from the mirVana　miRNA mimic panel (Ambion), was used to transfect the TE2, TE5, TE8, KYSE150, and KYSE790 cells at a final concentration of 12 μM by using Lipofectamine RNAiMAX (Invitrogen) according to the manufacturer’s instructions. After 72 h, the overexpression of miR-655 was confirmed by qRT-PCR using a Human TaqMan MicroRNA Assay Kit (Applied Biosystems).

***Proliferation assay and cell cycle analysis***

To measure cell growth, the number of viable cells at various time points after transfection was assessed by the colorimetric water-soluble tetrazolium salt assay (Cell Counting Kit 8; Dojindo Laboratories, Kumamoto, Japan). Cell viability was determined by reading the optical density at 450 nm. The cell cycle was evaluated 72h after transfection by fluorescence-activated cell sorting (FACS), as described elsewhere [12]. For the FACS analysis, harvested cells were fixed in 70% cold ethanol and treated with RNase A and propidium iodide. Samples were analyzed on a Becton Dickinson Accuri^TM^ C6 Flow Cytometer (Becton Dickinson, San Jose, CA).

***Trans-well migration and invasion assays***

Transwell migration and invasion assays were conducted in 24-well modified Boyden chambers (Transwell chambers, BD Transduction, Franklin Lakes, NJ). The upper surface of 6.4-mm-diameter filters with 8-µm pores was precoated with (invasion assay) or without (migration assay) Matrigel (BD Transduction). The miRNA mimic transfectants (5 x 10^5^ cells per well) were transferred into the upper chamber. Following 22 h of incubation, the migrated or invasive cells on the lower surface of the filters were fixed and stained with Diff-Quik stain (Sysmex, Kobe, Japan), and stained cell nuclei were counted directly in triplicate.

***Western blot analysis***

Anti-ACTB, anti-p21, anti-PTEN, anti-E-cadherin, anti-Vimentin, anti-Snail, and anti-ZEB1 antibodies were purchased from Cell Signaling Technology (Cell Signaling Technology, USA). Cells were lysed, and their proteins were extracted using M-PER Mammalian^®^ Protein Extraction Reagent (Thermo Scientific, USA).

To select the most appropriate ESCC cell line for the experiment to assess whether miR-655 would suppress EMT, we confirmed the expression levels of EMT-associated mRNA, such as E-cadherin and Vimentin, in all ESCC cell lines. From the expression profiles of these miRNAs in 8 ESCC cells, we selected the TE8 cells, for which Vimentin mRNA expression was high and E-cadherin mRNA expression was low, as a further assay (**Supplementary Figure S4**).

***Isolation of exosomes from plasma and miRNA isolation from exosomes***

Exosomes were extracted through proteinase K treatment from 100 μL of plasma using a Total Exosome RNA & Protein Isolation Kit (Invitrogen, USA) and subsequently resuspended to 25 μL in PBS. miRNAs were isolated from exosomes using a mirVana PARIS Kit (Ambion, Austin, TX) and were eluted into 100 μL of heated elution solution according to the manufacturer’s protocol.

***Animal experiment protocol***

The animal protocol was approved by the Institutional Animal Care and Use Committee of Kyoto Prefectural University of Medicine, and all experiments were conducted strictly in accordance to the National Institute of Health Guide for Care and Use of Laboratory Animals. To analyze the therapeutic effect of miR-655 for lymph node metastasis, we used the popliteal lymph node metastasis model for the *in vivo* analysis [13] [14]. Four-week-old BALB/c nude mice (SHIMIZU Laboratory Supplies, Kyoto, Japan) were used in this study. ESCC cells (2.5 × 10^6^ KYSE790) were inoculated to the right rear footpads of SCID mice. Treatment began at 7 days after tumor cell implantation. Either 1 nmol of the control miRNA mimic or 1 nmol of the miR-655 mimic with AteloGene Local Use Quick gelation (Koken, Co., Tokyo, Japan) was subcutaneously injected into the ventral surface of the lower flank region, which is far from the region injected with cancer cells, according to the manufacturer’s protocol. These treatments were continued every seven days for three weeks. The tumor volume was calculated according to the formula V = A × B^2^ / 2 (mm^3^), where A is the largest diameter (mm), and B is the smallest diameter (mm). At 22 days after tumor cell implantation, the mice were sacrificed, and each sample, such as the blood sample, tumor tissue, and popliteal lymph node sample, was collected for further analysis. For the analysis of lymph node metastasis, resected popliteal lymph nodes were fixed with 10% formaldehyde in PBS, embedded in paraffin. We sectioned each popliteal lymph node into three 5-μm-thick slices and evaluated the presence of lymph node metastasis by hematoxylin–eosin staining. Lymph node metastasis was diagnosed by a pathologist.

***Statistical analysis***

The Mann–Whitney U test and the Student's t-test for unpaired data were used to compare the plasma or tissue sample data. The Wilcoxon test was used to compare the paired tumor and normal tissue samples. The Chi-square test or Fisher’s exact probability test was used to evaluate correlations between the plasma miR-655 level and clinicopathological factors. The analysis was performed by dividing the patients into two groups using the median relative expression of plasma miR-655, 7.42, in ESCC patients as a cut-off. A *P*-value < 0.05 was considered statistically significant. Receiver-operating characteristic (ROC) curves and area under the ROC curve (AUC) values were used to assess the feasibility of using plasma miRNA levels as a diagnostic tool for detecting ESCC. The ROC curve was created by plotting the sensitivity against the false positive rate (1 - specificity) at various threshold settings. The Youden index was used to determine the cut-off value for the plasma miRNA levels [15]. For the survival rate analysis, Kaplan–Meier survival curves were constructed for groups based on univariate predictors, and differences between the groups were analyzed with the log-rank test. Univariate and multivariate survival analyses were performed using the likelihood ratio test of the stratified Cox proportional hazards model. A *P*-value < 0.05 was considered statistically significant.

**Supplementary references**

1. Rice TW, Ishwaran H, Hofstetter WL, Kelsen DP, Apperson-Hansen C, Blackstone EH: **Recommendations for pathologic staging (pTNM) of cancer of the esophagus and esophagogastric junction for the 8th edition AJCC/UICC staging manuals.** *Dis Esophagus* 2016, **29:**897-905.

2. Tsujiura M, Ichikawa D, Komatsu S, Shiozaki A, Takeshita H, Kosuga T, Konishi H, Morimura R, Deguchi K, Fujiwara H, et al: **Circulating microRNAs in plasma of patients with gastric cancers.** *Br J Cancer* 2010, **102:**1174-1179.

3. Komatsu S, Ichikawa D, Takeshita H, Tsujiura M, Morimura R, Nagata H, Kosuga T, Iitaka D, Konishi H, Shiozaki A, et al: **Circulating microRNAs in plasma of patients with oesophageal squamous cell carcinoma.** *Br J Cancer* 2011, **105:**104-111.

4. Morimura R, Komatsu S, Ichikawa D, Takeshita H, Tsujiura M, Nagata H, Konishi H, Shiozaki A, Ikoma H, Okamoto K, et al: **Novel diagnostic value of circulating miR-18a in plasma of patients with pancreatic cancer.** *Br J Cancer* 2011, **105:**1733-1740.

5. Konishi H, Ichikawa D, Komatsu S, Shiozaki A, Tsujiura M, Takeshita H, Morimura R, Nagata H, Arita T, Kawaguchi T, et al: **Detection of gastric cancer-associated microRNAs on microRNA microarray comparing pre- and post-operative plasma.** *Br J Cancer* 2012, **106:**740-747.

6. Kawaguchi T, Komatsu S, Ichikawa D, Morimura R, Tsujiura M, Konishi H, Takeshita H, Nagata H, Arita T, Hirajima S, et al: **Clinical impact of circulating miR-221 in plasma of patients with pancreatic cancer.** *Br J Cancer* 2013, **108:**361-369.

7. Hirajima S, Komatsu S, Ichikawa D, Takeshita H, Konishi H, Shiozaki A, Morimura R, Tsujiura M, Nagata H, Kawaguchi T, et al: **Clinical impact of circulating miR-18a in plasma of patients with oesophageal squamous cell carcinoma.** *Br J Cancer* 2013, **108:**1822-1829.

8. Komatsu S, Ichikawa D, Hirajima S, Kawaguchi T, Miyamae M, Okajima W, Ohashi T, Arita T, Konishi H, Shiozaki A, et al: **Plasma microRNA profiles: identification of miR-25 as a novel diagnostic and monitoring biomarker in oesophageal squamous cell carcinoma.** *Br J Cancer* 2014, **111:**1614-1624.

9. Mitchell PS, Parkin RK, Kroh EM, Fritz BR, Wyman SK, Pogosova-Agadjanyan EL, Peterson A, Noteboom J, O'Briant KC, Allen A, et al: **Circulating microRNAs as stable blood-based markers for cancer detection.** *Proc Natl Acad Sci U S A* 2008, **105:**10513-10518.

10. Livak KJ, Schmittgen TD: **Analysis of relative gene expression data using real-time quantitative PCR and the 2(-Delta Delta C(T)) Method.** *Methods* 2001, **25:**402-408.

11. Pfaffl MW: **A new mathematical model for relative quantification in real-time RT-PCR.** *Nucleic Acids Res* 2001, **29:**e45.

12. Komatsu S, Imoto I, Tsuda H, Kozaki KI, Muramatsu T, Shimada Y, Aiko S, Yoshizumi Y, Ichikawa D, Otsuji E, Inazawa J: **Overexpression of SMYD2 relates to tumor cell proliferation and malignant outcome of esophageal squamous cell carcinoma.** *Carcinogenesis* 2009, **30:**1139-1146.

13. Ito T, Shimada Y, Kan T, David S, Cheng Y, Mori Y, Agarwal R, Paun B, Jin Z, Olaru A, et al: **Pituitary tumor-transforming 1 increases cell motility and promotes lymph node metastasis in esophageal squamous cell carcinoma.** *Cancer Res* 2008, **68:**3214-3224.

14. Liu L, Lin C, Liang W, Wu S, Liu A, Wu J, Zhang X, Ren P, Li M, Song L: **TBL1XR1 promotes lymphangiogenesis and lymphatic metastasis in esophageal squamous cell carcinoma.** *Gut* 2015, **64:**26-36.

15. Akobeng AK: **Understanding diagnostic tests 3: Receiver operating characteristic curves.** *Acta Paediatr* 2007, **96:**644-647.
