# Supplementary figures and images for: Low levels of tumour suppressor miR-655 in plasma contribute to lymphatic progression and poor outcomes in oesophageal squamous cell carcinoma
Source: Mol Cancer. 2019 Jan 4;18:2. doi: 10.1186/s12943-018-0929-3 (PMC6320607; doi:10.1186/s12943-018-0929-3)

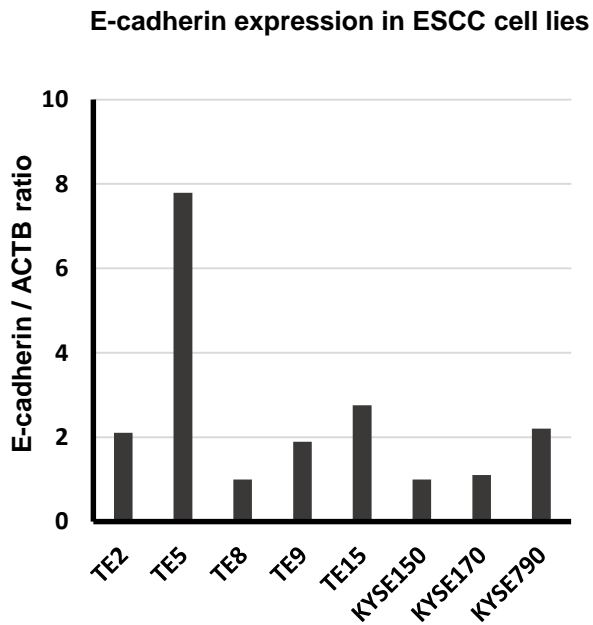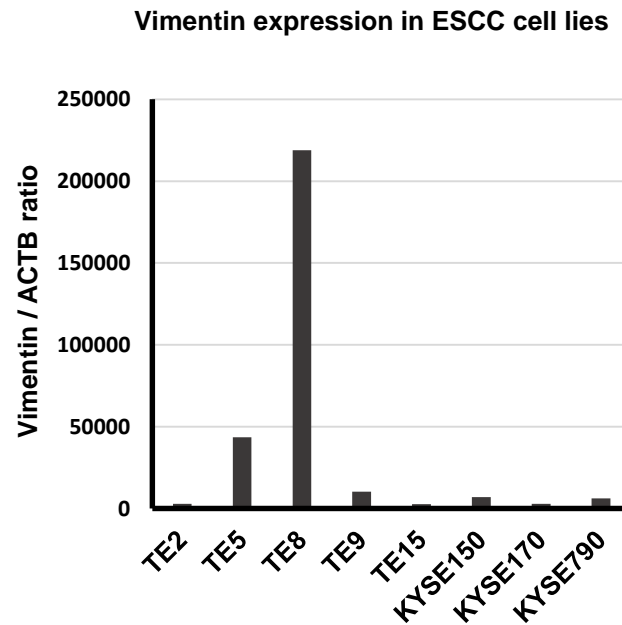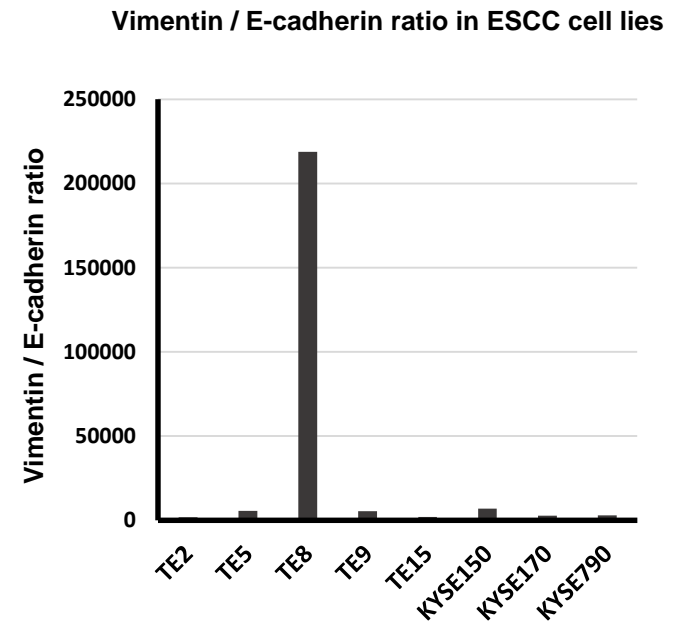

**Supplementary Figure S4.**

Supplement: Supplementary file 2 — Figure S4. Identification of the appropriate ESCC cell lines for investigation into whether miR-655 can suppress EMT. The panel mRNA expression levels of E-cadherin and Vimentin were investigated, and the Vimentin / E-cadherin ratio was calculated. In TE8 cells, Vimentin mRNA expression was high and E-cadherin mRNA expression was low. (PDF 85 kb) [file 12943_2018_929_MOESM2_ESM.pdf]

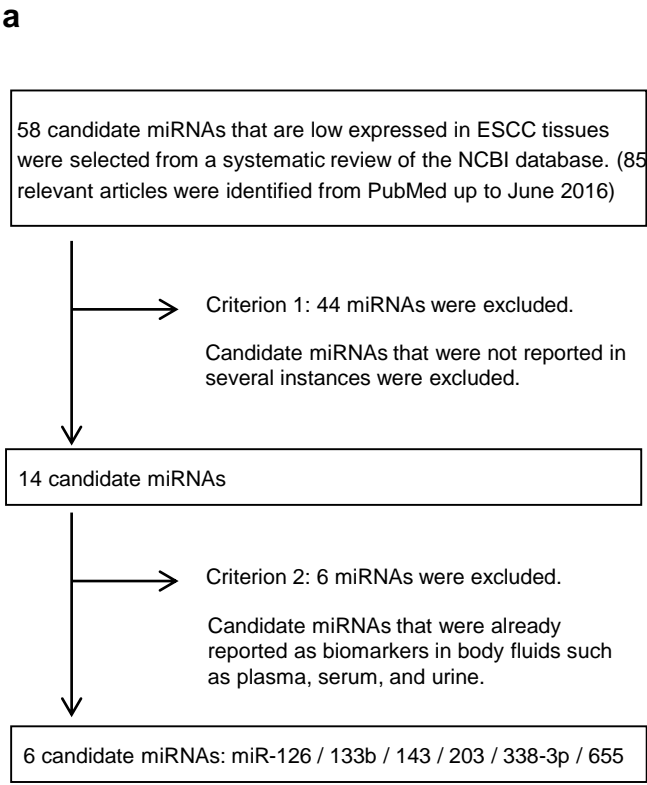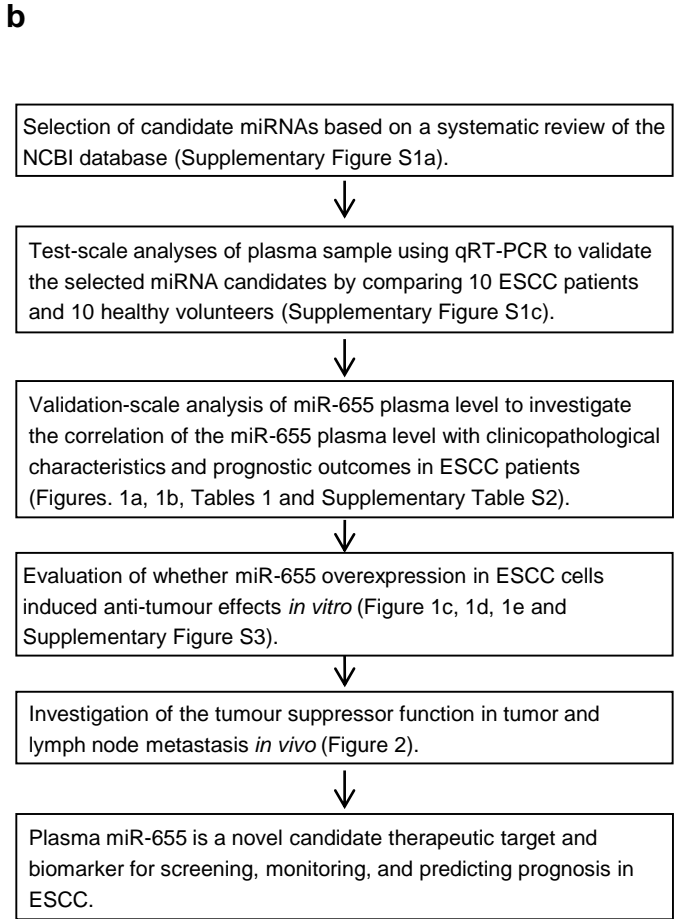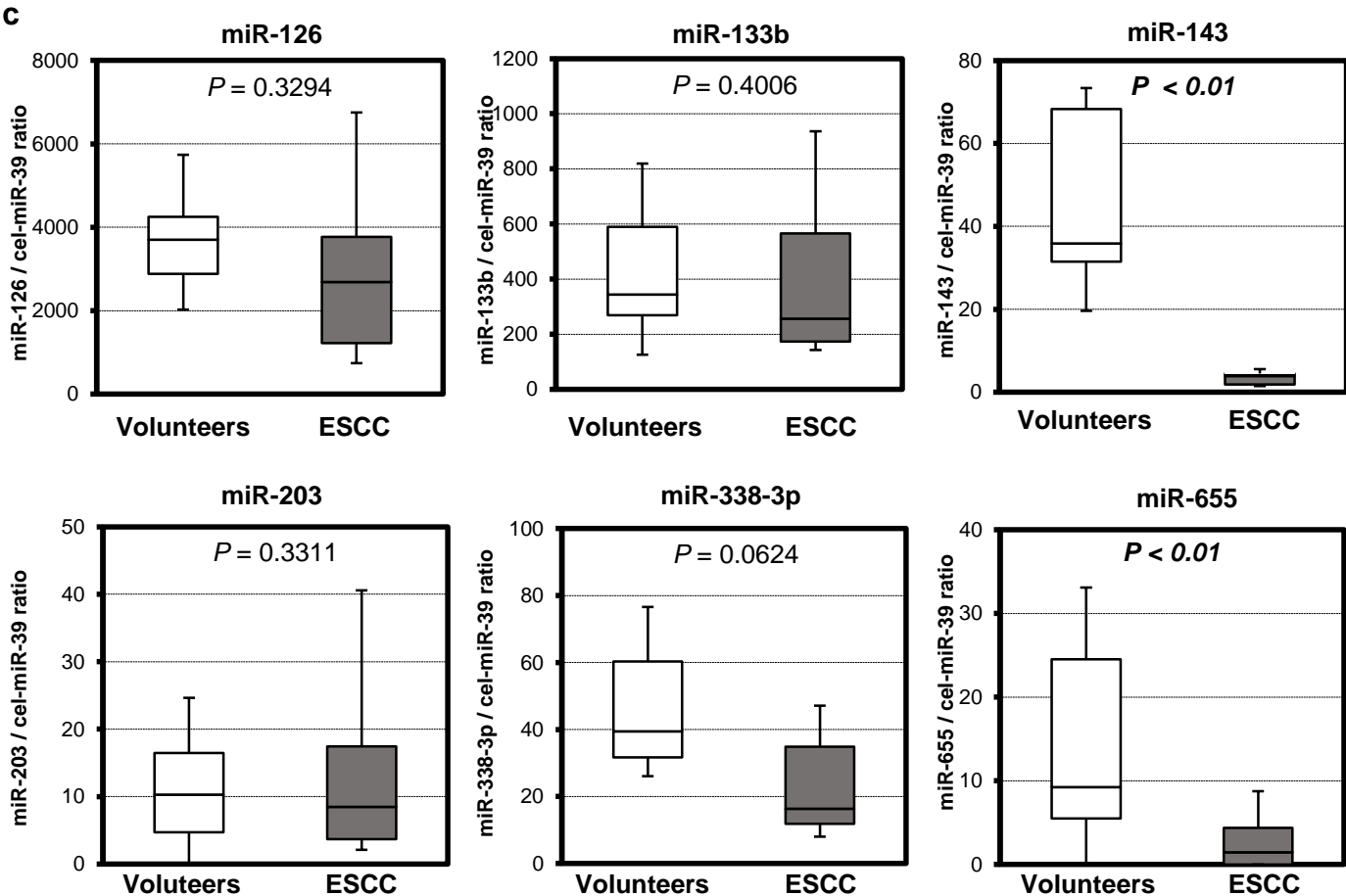

Supplementary Figure S1.

Supplement: Supplementary file 5 — Figure S1. Study design and selection of plasma miRNA candidates. (a) A systematic review of the NCBI database to identify novel plasma biomarkers of miRNA in patients with ESCC. This study was designed as follows: (1) selection of candidate miRNAs based on a systematic review of the NCBI database; (2) test-scale analysis of plasma samples using qRT-PCR to validate the utility of the selected miRNA candidates; (3) validation-scale analysis of the miR-655 plasma levels to investigate the correlations of the miR-655 plasma levels with clinicopathological characteristics and prognostic outcomes in ESCC patients; (4) evaluation of whether miR-655 overexpression in ESCC cells induced anti-tumour effects in vitro; and (5) investigation of the tumour suppressive function in tumour and lymph node metastasis in vivo. (b) Study design to find novel candidate miRNAs that decrease in patient plasma as a therapeutic target for ESCC. After a series of exclusion criteria were applied, we consequently selected six candidate miRNAs: miR-126, 133b, 143, 203, 338-3p, and 655. (c) Small-scale analysis of the plasma levels of six miRNAs in ESCC patients and healthy volunteers by qRT-PCR. We investigated the plasma levels of the six selected miRNAs in 10 ESCC patients and 10 healthy volunteers by qRT-PCR. The plasma levels of miR-143 and miR-655 were significantly down-regulated in ESCC patients compared to healthy volunteers, and we selected miR-655 for further analysis. (PDF 30 kb) [file 12943_2018_929_MOESM5_ESM.pdf]

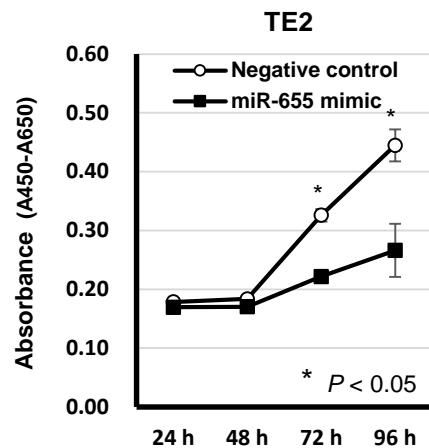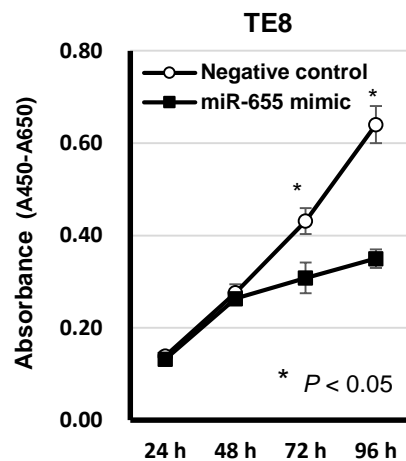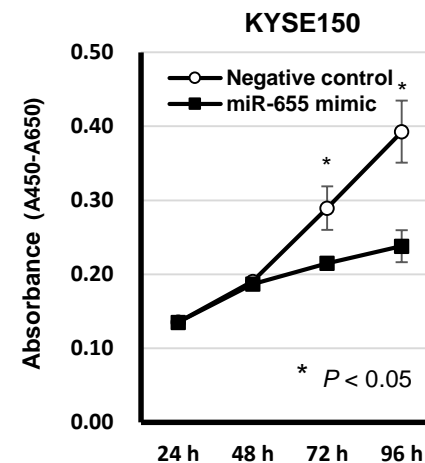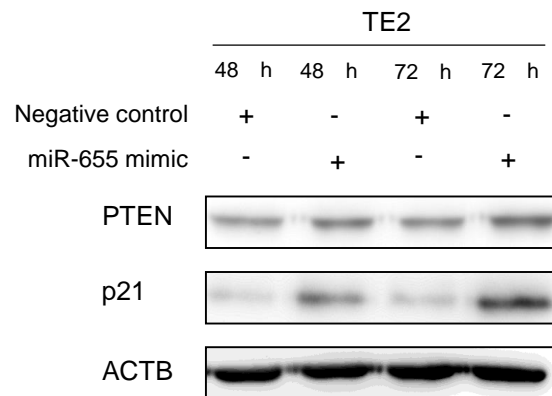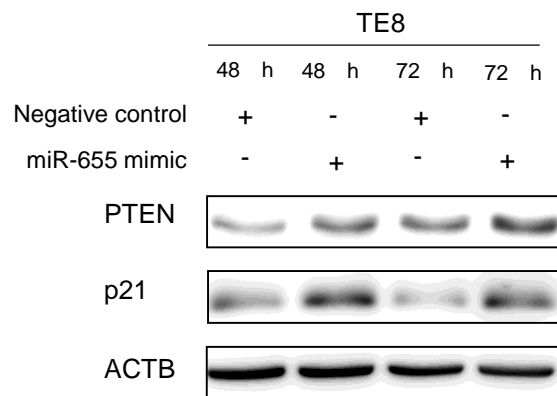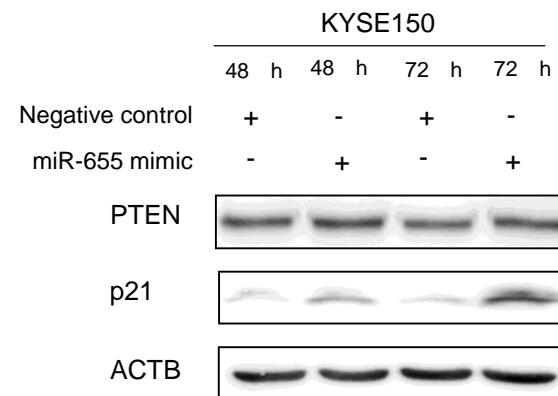

**Supplementary Figure S3.**

Supplement: Supplementary file 12 — Figure S3. Investigation into the tumour suppressive function of miR-655 in ESCC cells. Overexpression of miR-655 by miR-655 mimic induced the expression of p21 and PTEN proteins and significantly suppressed cell proliferation in TE2, TE8, and KYSE150 cells. (PDF 79 kb) [file 12943_2018_929_MOESM12_ESM.pdf]
