# Supplementary material for: Low levels of tumour suppressor miR-655 in plasma contribute to lymphatic progression and poor outcomes in oesophageal squamous cell carcinoma
Source: Mol Cancer. 2019 Jan 4;18:2. doi: 10.1186/s12943-018-0929-3 (PMC6320607; doi:10.1186/s12943-018-0929-3)
Supplement: Supplementary file 3 — Table S6. Clinical data of ESCC patients. (DOCX 47 kb) [file 12943_2018_929_MOESM3_ESM.docx]

**Additional file 3: Table S6.**

Clinical data of ESCC patients

| **Sample ID** | **Sex** | **Age** | **pT TNM** | **pN TNM** | **pStage TNM** | **ly** | **v** | **Tumor size (mm)** | **Histology** | **Cancer-related death** | **Days after surgery** | **Recurrence** |
| --- | --- | --- | --- | --- | --- | --- | --- | --- | --- | --- | --- | --- |
| ESCC 1 | M | 71 | T2 | N0 | II | 0 | 0 | 82 | well and moderately | Death | 317 | Present |
| ESCC 2 | M | 75 | T3 | N1 | III | 1 | 1 | 65 | well and moderately | Death | 221 | Present |
| ESCC 3 | M | 59 | T1 | N1 | II | 0 | 2 | 27 | poorly | Alive | 2000 | Absent |
| ESCC 4 | M | 41 | T3 | N1 | III | 0 | 1 | 125 | well and moderately | Alive | 1945 | Absent |
| ESCC 5 | M | 75 | T2 | N2 | III | 3 | 0 | 46 | well and moderately | Death | 760 | Present |
| ESCC 6 | M | 63 | T3 | N2 | III | 1 | 1 | 55 | well and moderately | Death | 588 | Present |
| ESCC 7 | M | 63 | T3 | N1 | III | 1 | 2 | 55 | well and moderately | Death | 741 | Present |
| ESCC 8 | M | 67 | T2 | N1 | II | 0 | 0 | 40 | poorly | Death | 1318 | Present |
| ESCC 9 | F | 49 | T2 | N2 | III | 3 | 0 | 105 | well and moderately | Death | 266 | Present |
| ESCC 10 | M | 76 | T2 | N2 | III | 0 | 2 | 12 | poorly | Alive | 1421 | Absent |
| ESCC 11 | M | 63 | T3 | N1 | III | 0 | 1 | 34 | well and moderately | Alive | 1997 | Present |
| ESCC 12 | F | 70 | T3 | N2 | III | 1 | 2 | 29 | well and moderately | Death | 525 | Present |
| ESCC 13 | M | 72 | T4 | N1 | III | 1 | 1 | 105 | poorly | Death | 1319 | Absent |
| ESCC 14 | M | 77 | T3 | N1 | III | 2 | 0 | 35 | poorly | Alive | 650 | Absent |
| ESCC 15 | M | 61 | T3 | N2 | III | 2 | 0 | 50 | well and moderately | Death | 1078 | Present |
| ESCC 16 | M | 73 | T1 | N1 | II | 2 | 1 | 40 | well and moderately | Alive | 1690 | Absent |
| ESCC 17 | F | 68 | T3 | N1 | III | 1 | 3 | 56 | well and moderately | Alive | 377 | Present |
| ESCC 18 | M | 79 | T1 | N0 | I | 0 | 2 | 1 | well and moderately | Alive | 68 | Absent |
| ESCC 19 | M | 57 | T2 | N1 | II | 0 | 1 | 25 | poorly | Death | 388 | Present |
| ESCC 20 | M | 57 | T1 | N0 | I | 0 | 0 | 90 | poorly | Alive | 2000 | Absent |
| ESCC 21 | M | 68 | T3 | N2 | III | 3 | 1 | 40 | poorly | Death | 348 | Present |
| ESCC 22 | M | 57 | T3 | N2 | III | 0 | 0 | 12 | well and moderately | Alive | 2000 | Absent |
| ESCC 23 | M | 55 | T1 | N0 | I | 0 | 0 | 42 | poorly | Alive | 2000 | Absent |
| ESCC 24 | M | 67 | T2 | N1 | II | 1 | 2 | 33 | well and moderately | Alive | 1377 | Absent |
| ESCC 25 | M | 67 | T3 | N0 | II | 0 | 1 | 50 | well and moderately | Death | 171 | Present |
| ESCC 26 | F | 61 | T3 | N0 | II | 0 | 0 | 25 | well and moderately | Alive | 2000 | Absent |
| ESCC 27 | M | 67 | T4 | N3 | III | 2 | 2 | 20 | well and moderately | Alive | 90 | Absent |
| ESCC 28 | M | 88 | T3 | N1 | III | 1 | 3 | 55 | poorly | Alive | 637 | Present |
| ESCC 29 | M | 68 | T1 | N1 | II | 1 | 0 | 40 | well and moderately | Death | 548 | Present |
| ESCC 30 | F | 52 | T1 | N0 | I | 0 | 0 | 5 | well and moderately | Alive | 554 | Absent |
| ESCC 31 | M | 69 | T3 | N2 | III | 1 | 1 | 110 | well and moderately | Alive | 279 | Absent |
| ESCC 32 | M | 66 | T1 | N1 | II | 0 | 0 | 5 | well and moderately | Alive | 36 | Absent |
| ESCC 33 | M | 68 | T3 | N1 | III | 1 | 1 | 40 | well and moderately | Alive | 241 | Absent |
| ESCC 34 | F | 60 | T3 | N1 | III | 1 | 1 | 60 | well and moderately | Alive | 2000 | Absent |
| ESCC 35 | F | 58 | T2 | N0 | II | 1 | 1 | 60 | well and moderately | Alive | 379 | Absent |
| ESCC 36 | M | 57 | T3 | N1 | III | 1 | 1 | 33 | well and moderately | Alive | 1835 | Absent |
| ESCC 37 | F | 61 | T3 | N0 | II | 1 | 0 | 38 | well and moderately | Alive | 1833 | Present |
| ESCC 38 | M | 61 | T4 | N0 | III | 1 | 1 | 58 | well and moderately | Death | 284 | Present |
| ESCC 39 | M | 74 | T3 | N1 | III | 0 | 2 | 55 | well and moderately | Alive | 1987 | Absent |
| ESCC 40 | F | 70 | T1 | N0 | I | 0 | 2 | 40 | well and moderately | Alive | 134 | Absent |
| ESCC 41 | M | 76 | T3 | N1 | III | 1 | 3 | 45 | poorly | Alive | 312 | Absent |
| ESCC 42 | M | 84 | T2 | N2 | III | 1 | 1 | 82 | well and moderately | Alive | 1919 | Present |
| ESCC 43 | F | 79 | T3 | N1 | III | 1 | 3 | 55 | well and moderately | Death | 705 | Present |
| ESCC 44 | F | 74 | T3 | N3 | III | 1 | 1 | 130 | well and moderately | Alive | 85 | Absent |
| ESCC 45 | M | 74 | T3 | N1 | III | 3 | 3 | 60 | poorly | Death | 89 | Absent |
| ESCC 46 | M | 64 | T3 | N1 | III | 0 | 1 | 46 | well and moderately | Alive | 24 | Absent |
| ESCC 47 | F | 82 | T3 | N1 | III | 2 | 3 | 70 | poorly | Alive | 319 | Present |
| ESCC 48 | M | 62 | T2 | N1 | II | 0 | 1 | 25 | well and moderately | Alive | 1819 | Absent |
| ESCC 49 | F | 70 | T2 | N2 | III | 2 | 0 | 90 | well and moderately | Alive | 1784 | Absent |
| ESCC 50 | M | 60 | T3 | N1 | III | 1 | 1 | 80 | well and moderately | Alive | 1024 | Present |
| ESCC 51 | M | 74 | T1 | N3 | III | 0 | 0 | 84 | poorly | Death | 718 | Present |
| ESCC 52 | F | 55 | T1 | N0 | I | 0 | 0 | 18 | poorly | Alive | 328 | Absent |
| ESCC 53 | M | 70 | T3 | N1 | III | 3 | 1 | 77 | well and moderately | Death | 5 | Absent |
| ESCC 54 | M | 62 | T3 | N1 | III | 1 | 3 | 70 | well and moderately | Death | 144 | Present |
| ESCC 55 | M | 66 | T3 | N1 | III | 0 | 0 | 5 | well and moderately | Alive | 1814 | Absent |
| ESCC 56 | M | 61 | T1 | N1 | II | 1 | 0 | 10 | poorly | Alive | 1772 | Absent |
| ESCC 57 | F | 63 | T4 | N2 | III | 1 | 1 | 60 | well and moderately | Death | 234 | Present |
| ESCC 58 | M | 49 | T3 | N1 | III | 1 | 3 | 55 | well and moderately | Death | 351 | Absent |
| ESCC 59 | F | 71 | T2 | N2 | III | 0 | 1 | 40 | well and moderately | Death | 1542 | Present |
| ESCC 60 | F | 52 | T3 | N1 | III | 3 | 0 | 40 | well and moderately | Alive | 2000 | Present |
| ESCC 61 | M | 67 | T3 | N2 | III | 2 | 0 | 54 | well and moderately | Alive | 1632 | Absent |
| ESCC 62 | M | 69 | T3 | N1 | III | 1 | 0 | 25 | poorly | Alive | 1725 | Absent |
| ESCC 63 | M | 64 | T1 | N0 | I | 0 | 0 | 40 | well and moderately | Alive | 312 | Absent |
| ESCC 64 | M | 76 | T1 | N0 | I | 1 | 1 | 18 | well and moderately | Alive | 351 | Absent |
| ESCC 65 | M | 77 | T1 | N0 | I | 2 | 0 | 13 | well and moderately | Alive | 1721 | Present |
| ESCC 66 | M | 71 | T1 | N1 | II | 3 | 1 | 36 | well and moderately | Alive | 1667 | Absent |
| ESCC 67 | M | 69 | T3 | N1 | III | 1 | 0 | 56 | well and moderately | Alive | 159 | Absent |
| ESCC 68 | M | 60 | T3 | N1 | III | 3 | 0 | 30 | well and moderately | Alive | 1651 | Absent |
| ESCC 69 | M | 75 | T1 | N2 | II | 1 | 0 | 25 | well and moderately | Alive | 1511 | Absent |
| ESCC 70 | M | 72 | T1 | N1 | II | 0 | 0 | 3 | well and moderately | Death | 701 | Present |
| ESCC 71 | M | 66 | T3 | N1 | III | 0 | 1 | 75 | well and moderately | Death | 472 | Present |
| ESCC 72 | F | 61 | T3 | N2 | III | 2 | 1 | 30 | well and moderately | Alive | 1571 | Present |
| ESCC 73 | M | 72 | T3 | N1 | III | 2 | 2 | 55 | well and moderately | Death | 132 | Present |
| ESCC 74 | M | 62 | T3 | N3 | III | 3 | 1 | 63 | well and moderately | Alive | 810 | Present |
| ESCC 75 | M | 63 | T1 | N0 | I | 0 | 0 | 40 | well and moderately | Alive | 230 | Absent |
| ESCC 76 | M | 80 | T2 | N0 | II | 1 | 1 | 27 | well and moderately | Death | 113 | Absent |
| ESCC 77 | M | 79 | T1 | N1 | II | 0 | 0 | 4 | poorly | Death | 705 | Present |
| ESCC 78 | M | 77 | T2 | N2 | III | 0 | 1 | 36 | poorly | Death | 1283 | Present |
| ESCC 79 | M | 69 | T3 | N0 | II | 0 | 2 | 55 | well and moderately | Alive | 1602 | Absent |
| ESCC 80 | M | 70 | T3 | N0 | II | 0 | 2 | 24 | well and moderately | Alive | 1347 | Absent |
| ESCC 81 | M | 74 | T2 | N2 | III | 1 | 0 | 15 | poorly | Alive | 1518 | Absent |
| ESCC 82 | M | 75 | T3 | N0 | II | 0 | 1 | 35 | well and moderately | Death | 485 | Absent |
| ESCC 83 | M | 67 | T2 | N4 | IV | 3 | 3 | 150 | well and moderately | Death | 337 | Present |
| ESCC 84 | M | 69 | T3 | N3 | III | 0 | 0 | 5 | well and moderately | Alive | 6 | Absent |
| ESCC 85 | M | 74 | T3 | N1 | III | 1 | 0 | 50 | well and moderately | Alive | 383 | Present |
| ESCC 86 | M | 51 | T3 | N0 | II | 0 | 0 | 22 | well and moderately | Alive | 479 | Absent |
| ESCC 87 | M | 73 | T3 | N0 | II | 0 | 0 | 28 | well and moderately | Death | 316 | Present |
| ESCC 88 | M | 75 | T3 | N0 | II | 0 | 0 | 20 | well and moderately | Alive | 1480 | Absent |
| ESCC 89 | M | 57 | T1 | N0 | I | 0 | 0 | 40 | well and moderately | Alive | 32 | Absent |
| ESCC 90 | M | 51 | T2 | N2 | III | 0 | 2 | 40 | well and moderately | Alive | 721 | Absent |
| ESCC 91 | M | 79 | T2 | N1 | II | 2 | 1 | 40 | poorly | Death | 1021 | Present |
| ESCC 92 | M | 71 | T2 | N1 | II | 0 | 1 | 45 | well and moderately | Alive | 1536 | Absent |
| ESCC 93 | M | 75 | T1 | N3 | III | 3 | 3 | 40 | poorly | Death | 271 | Present |
| ESCC 94 | M | 68 | T3 | N0 | II | 1 | 1 | 60 | well and moderately | Alive | 1251 | Absent |
| ESCC 95 | M | 65 | T3 | N1 | III | 2 | 0 | 40 | poorly | Alive | 1821 | Present |
| ESCC 96 | F | 48 | T3 | N0 | II | 0 | 2 | 60 | well and moderately | Alive | 1491 | Absent |
| ESCC 97 | F | 69 | T3 | N2 | III | 1 | 1 | 44 | well and moderately | Death | 421 | Present |
| ESCC 98 | M | 65 | T2 | N0 | II | 0 | 2 | 60 | well and moderately | Alive | 1521 | Absent |
| ESCC 99 | F | 63 | T1 | N1 | II | 1 | 1 | 40 | poorly | Death | 1192 | Present |
| ESCC 100 | M | 70 | T2 | N1 | II | 2 | 0 | 60 | well and moderately | Death | 672 | Present |
| ESCC 101 | M | 69 | T1 | N0 | I | 1 | 0 | 60 | well and moderately | Alive | 1901 | Absent |
| ESCC 102 | M | 66 | T2 | N0 | II | 1 | 1 | 60 | well and moderately | Alive | 2000 | Absent |
| ESCC 103 | M | 68 | T3 | N0 | II | 0 | 2 | 60 | well and moderately | Alive | 2000 | Absent |
| ESCC 104 | M | 53 | T1 | N0 | I | 0 | 0 | 7 | poorly | Alive | 2000 | Absent |
| ESCC 105 | M | 68 | T3 | N1 | III | 3 | 1 | 40 | poorly | Death | 271 | Present |
| ESCC 106 | M | 63 | T2 | N3 | III | 1 | 1 | 60 | well and moderately | Death | 271 | Present |
| ESCC 107 | M | 42 | T1 | N0 | I | 0 | 2 | 40 | well and moderately | Alive | 421 | Absent |
| ESCC 108 | M | 63 | T2 | N1 | II | 3 | 1 | 60 | well and moderately | Alive | 278 | Present |
| ESCC 109 | M | 60 | T1 | N0 | I | 0 | 2 | 60 | well and moderately | Alive | 690 | Absent |
| ESCC 110 | F | 65 | T1 | N0 | I | 1 | 0 | 60 | well and moderately | Alive | 834 | Absent |
| ESCC 111 | M | 67 | T2 | N1 | II | 0 | 1 | 40 | poorly | Alive | 542 | Present |
| ESCC 112 | M | 70 | T2 | N2 | III | 2 | 1 | 60 | well and moderately | Alive | 521 | Absent |
| ESCC 113 | M | 70 | T2 | N3 | III | 3 | 1 | 40 | poorly | Death | 249 | Present |
| ESCC 114 | M | 66 | T1 | N0 | I | 1 | 0 | 60 | well and moderately | Alive | 2000 | Absent |
| ESCC 115 | M | 71 | T3 | N2 | III | 2 | 1 | 12 | poorly | Death | 1281 | Present |
| ESCC 116 | M | 49 | T1 | N1 | II | 2 | 1 | 40 | poorly | Death | 792 | Present |
| ESCC 117 | F | 58 | T1 | N2 | II | 1 | 2 | 40 | poorly | Death | 441 | Present |
| ESCC 118 | M | 57 | T2 | N2 | III | 2 | 1 | 60 | well and moderately | Death | 621 | Present |
| ESCC 119 | M | 72 | T1 | N0 | I | 2 | 0 | 60 | well and moderately | Alive | 2000 | Absent |
| ESCC 120 | M | 62 | T2 | N1 | II | 0 | 2 | 60 | well and moderately | Alive | 2000 | Absent |
| ESCC 121 | M | 68 | T3 | N1 | III | 1 | 1 | 60 | well and moderately | Alive | 1037 | Absent |
| ESCC 122 | M | 49 | T2 | N1 | II | 2 | 1 | 60 | well and moderately | Alive | 1745 | Absent |
