# Supplementary material for: Low levels of tumour suppressor miR-655 in plasma contribute to lymphatic progression and poor outcomes in oesophageal squamous cell carcinoma
Source: Mol Cancer. 2019 Jan 4;18:2. doi: 10.1186/s12943-018-0929-3 (PMC6320607; doi:10.1186/s12943-018-0929-3)
Supplement: Supplementary file 4 — Table S7. The primer sequences used in this study. (DOCX 16 kb) [file 12943_2018_929_MOESM4_ESM.docx]

**Additional file 4: Table S7.**

The primer sequences used in this study.

| **Primer** | **Assay ID** | **Sequence** |
| --- | --- | --- |
| hsa-miR-126 | 002228 | UCGUACCGUGAGUAAUAAUGCG |
| hsa-miR-133b | 002247 | UUUGGUCCCCUUCAACCAGCUA |
| hsa-miR-143 | 002249 | UGAGAUGAAGCACUGUAGCUC |
| hsa-miR-203 | 000507 | GUGAAAUGUUUAGGACCACUAG |
| hsa-miR-338-3p | 002252 | UCCAGCAUCAGUGAUUUUGUUG |
| hsa-miR-655 | 001612 | AUAAUACAUGGUUAACCUCUUU |
| cel-miR-39 | 000200 | UCACCGGGUGUAAAUCAGCUUG |
