# Supplementary material for: Low levels of tumour suppressor miR-655 in plasma contribute to lymphatic progression and poor outcomes in oesophageal squamous cell carcinoma
Source: Mol Cancer. 2019 Jan 4;18:2. doi: 10.1186/s12943-018-0929-3 (PMC6320607; doi:10.1186/s12943-018-0929-3)
Supplement: Supplementary file 6 — Table S1. Selected process of all candidate miRnas. (PDF 55 kb) [file 12943_2018_929_MOESM6_ESM.pdf]

Supplementary Table S1.

Candidate miRNA	Reported function in tissue	Criterion 1 (sufficient data)	Criterion 2 (not reported in body fluids)
let-7	tumour-suppressor	negative	already excluded
let-7a	tumour-suppressor	negative	already excluded
let-7b	tumour-suppressor	negative	already excluded
let-7c	tumour-suppressor	negative	already excluded
let-7g	tumour-suppressor	negative	already excluded
let-7i	tumour-suppressor	negative	already excluded
miR-1	tumour-suppressor	negative	already excluded
miR-10a	tumour-suppressor	negative	already excluded
miR-22	tumour-suppressor	negative	already excluded
miR-27a	tumour-suppressor	negative	already excluded
miR-29b	tumour-suppressor	negative	already excluded
miR-29c	tumour-suppressor	positive	negative
miR-34a	tumour-suppressor	negative	already excluded
miR-92b	tumour-suppressor	negative	already excluded
miR-98	tumour-suppressor	negative	already excluded
miR-99a	tumour-suppressor	negative	already excluded
miR-100	tumour-suppressor	positive	negative
miR-101	tumour-suppressor	negative	already excluded
miR-106a	tumour-suppressor	negative	already excluded
miR-107	tumour-suppressor	negative	already excluded
miR-126	tumour-suppressor	positive	positive
miR-129-5p	tumour-suppressor	negative	already excluded
miR-133a	tumour-suppressor	positive	negative
miR-133b	tumour-suppressor	positive	positive
miR-138	tumour-suppressor	negative	already excluded
miR-139-5p	tumour-suppressor	negative	already excluded
miR-143	tumour-suppressor	positive	positive
miR-145	tumour-suppressor	positive	negative
miR-150	tumour-suppressor	negative	already excluded
miR-185	tumour-suppressor	positive	negative
miR-195	tumour-suppressor	negative	already excluded

miR-200b	tumour-suppressor	negative	already excluded
miR-202	tumour-suppressor	negative	already excluded
miR-203	tumour-suppressor	positive	positive
miR-205	tumour-suppressor	negative	already excluded
miR-210	tumour-suppressor	negative	already excluded
miR-214	tumour-suppressor	negative	already excluded
miR-217	tumour-suppressor	negative	already excluded
miR-218	tumour-suppressor	negative	already excluded
miR-302b	tumour-suppressor	negative	already excluded
miR-335	tumour-suppressor	negative	already excluded
miR-338-3p	tumour-suppressor	positive	positive
miR-3651	tumour-suppressor	negative	already excluded
miR-375	tumour-suppressor	positive	negative
miR-382	tumour-suppressor	negative	already excluded
miR-450a	tumour-suppressor	negative	already excluded
miR-451	tumour-suppressor	negative	already excluded
miR-451	tumour-suppressor	negative	already excluded
miR-494	tumour-suppressor	negative	already excluded
miR-507	tumour-suppressor	negative	already excluded
miR-518b	tumour-suppressor	negative	already excluded
miR-520a	tumour-suppressor	negative	already excluded
miR-542-3p	tumour-suppressor	negative	already excluded
miR-634	tumour-suppressor	negative	already excluded
miR-655	tumour-suppressor	positive	positive
miR-720	tumour-suppressor	negative	already excluded
miR-1274a	tumour-suppressor	negative	already excluded
miR-1291	tumour-suppressor	negative	already excluded

Supplementary Table S1.

Selected process of all candidate miRNAs
