# Supplementary material for: Low levels of tumour suppressor miR-655 in plasma contribute to lymphatic progression and poor outcomes in oesophageal squamous cell carcinoma
Source: Mol Cancer. 2019 Jan 4;18:2. doi: 10.1186/s12943-018-0929-3 (PMC6320607; doi:10.1186/s12943-018-0929-3)
Supplement: Supplementary file 7 — Figure S2. MiR-655 levels in plasma, normal human organs and cell lines. (a) Receiver-operating characteristic (ROC) curves and area under the ROC curve (AUC) values were used to assess the feasibility of using plasma miRNA levels as a diagnostic tool for detecting ESCC (AUC 0.782). The optimal relative expression cut-off point was found to be 26.57 using the miR-655 / cel-miR-39 ratio, with a sensitivity of 79.5% and a specificity of 67.3%. (b) miR-655 levels in normal human organs and ESCC cell lines. miR-655 was highly expressed in the brain, testis, colon, and oesophagus. In all ESCC cell lines, the expression of miR-655 was lower than in the normal oesophageal mucosa. (c) miR-655 levels in non-cancerous and ESCC tissues. The expression of miR-655 was significantly higher in the non-cancerous oesophageal mucosa than in ESCC tissues (P < 0.01). (d) miR-655 levels in exosomes extracted from the plasma of ESCC patients and healthy volunteers. The expression levels of miR-655 in exosomes extracted from plasma was investigated in 7 ESCC patients and 7 healthy volunteers. Exosomal miR-655 in plasma was significantly down-regulated in ESCC patients compared to healthy volunteers (P < 0.05). (PDF 89 kb) [file 12943_2018_929_MOESM7_ESM.pdf]

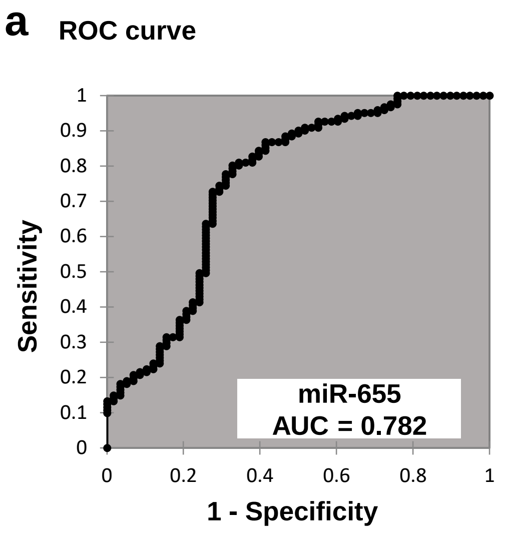

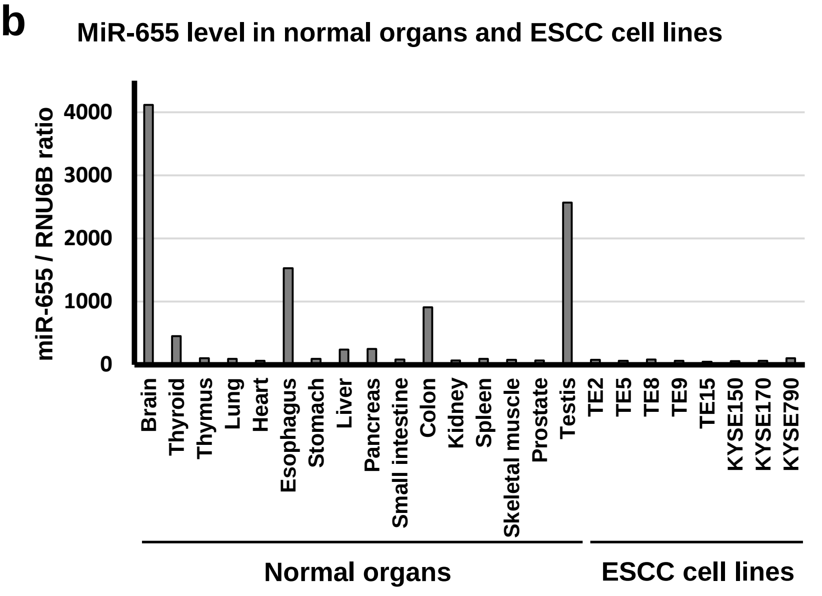

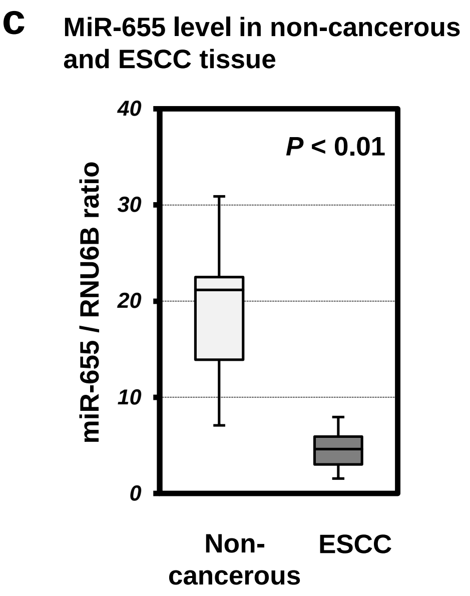

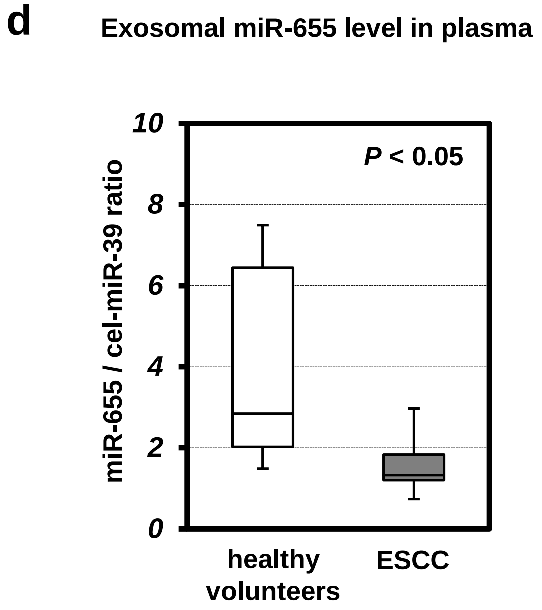

Supplementary Figure S2.
