# Supplementary material for: Low levels of tumour suppressor miR-655 in plasma contribute to lymphatic progression and poor outcomes in oesophageal squamous cell carcinoma
Source: Mol Cancer. 2019 Jan 4;18:2. doi: 10.1186/s12943-018-0929-3 (PMC6320607; doi:10.1186/s12943-018-0929-3)
Supplement: Supplementary file 8 — Table S2. Univariate and multivariate analysis of ESCC patient survival following esophagectomy using the Cox proportional hazards model. (DOCX 17 kb) [file 12943_2018_929_MOESM8_ESM.docx]

| **Variable** | |  | **Univariate^a^** |  | **Multivariate^b^** | |  |
| --- | --- | --- | --- | --- | --- | --- | --- |
|  |  | **n** | ***P*-value** |  | **HR ^c^** | **95% CI^d^** | ***P*-value** |
| Gender | Male vs. female | 99 vs. 23 | 0.994 |  |  |  |  |
| Age (60 years old) | 60 < vs. < 60 | 99 vs. 23 | 0.264 |  |  |  |  |
| T stage (TNM classification) | T3,T4 vs. T1,T2 | 60 vs. 62 | 0.627 |  |  |  |  |
| N stage (TNM classification) | N2,N3,N4 vs. N0,N1 | 33 vs. 89 | **0.016** |  | 2.63 | 1.14–3.88 | **0.014** |
| Lymphatic invasion ( ly ) | ly2,ly3 vs. ly0,ly1 | 33 vs. 89 | 0.146 |  |  |  |  |
| Venous invasion ( v ) | v2,v3 vs. v0,v1 | 30 vs. 92 | 0.867 |  |  |  |  |
| Plasma miR-655 level | Low vs. high | 61 vs 61 | **0.028** |  | 2.34 | 1.11–5.92 | **0.021** |

**Additional file 8: Table S2.**

Univariate and multivariate analysis of ESCC patient survival following esophagectomy using the Cox proportional hazards model.

^a^ Univariate survival analysis was performed using the Kaplan–Meier method; the significance was determined by log-rank test. ^b^ Multivariate survival analysis was performed using the Cox proportional hazards model. ^C^ HR: Hazard ratio ^D^, CI: Confidence interval. NOTE: significant values are in bold
