# Supplementary material for: Low levels of tumour suppressor miR-655 in plasma contribute to lymphatic progression and poor outcomes in oesophageal squamous cell carcinoma
Source: Mol Cancer. 2019 Jan 4;18:2. doi: 10.1186/s12943-018-0929-3 (PMC6320607; doi:10.1186/s12943-018-0929-3)
Supplement: Supplementary file 9 — Table S3. Association between plasma miR-655 levels and clinicopathological characteristics in ESCC patients who were pathologically classified into Stage I. (DOCX 19 kb) [file 12943_2018_929_MOESM9_ESM.docx]

|  | | **Plasma miR-655 concentration** | | | |  |
| --- | --- | --- | --- | --- | --- | --- |
| **Variables** | | **high** | **(n=13)** | **low** | **(n=5)** | ***P*-value^a^** |
| Gender | Female | 3 | (75%) | 1 | (25%) | 1.000 |
|  | Male | 10 | (71%) | 4 | (29%) |  |
| Age (60 years old) | < 60 | 7 | (100%) | 0 | (0%) | 0.101 |
|  | 60 < | 6 | (54%) | 5 | (46%) |  |
| Lymphatic invasion | ly0 | 11 | (92%) | 1 | (8%) | **0.021** |
|  | ly1,2,3 | 2 | (33%) | 4 | (67%) |  |
| Venous invasion | v0 | 9 | (69%) | 4 | (31%) | 1.000 |
|  | v1,2,3 | 4 | (80%) | 1 | (20%) |  |
| Tumor size | < 50 mm | 11 | (92%) | 1 | (8%) | **0.021** |
|  | 50 mm < | 2 | (33%) | 4 | (67%) |  |
| Histology | Well and moderately differentiated | 9 | (64%) | 5 | (36%) | 0.277 |
|  | Poorly differentiated | 4 | (100%) | 0 | (0%) |  |
| Recurrences | Absent | 13 | (76%) | 4 | (24%) | 0.277 |
|  | Present | 0 | (0%) | 1 | (100%) |  |

**Additional file 9: Table S3.**

Association between plasma miR-655 levels and clinicopathological characteristics in ESCC patients who were pathologically classified into Stage I.

^a^ Chi-square or Fisher tests. NOTE: significant values are in bold.
