# Supplementary material for: Low levels of tumour suppressor miR-655 in plasma contribute to lymphatic progression and poor outcomes in oesophageal squamous cell carcinoma
Source: Mol Cancer. 2019 Jan 4;18:2. doi: 10.1186/s12943-018-0929-3 (PMC6320607; doi:10.1186/s12943-018-0929-3)
Supplement: Supplementary file 10 — Table S4. Association between plasma miR-655 levels and clinicopathological characteristics in ESCC patients who were pathologically classified into Stage II and III. (DOCX 20 kb) [file 12943_2018_929_MOESM10_ESM.docx]

|  | | **Plasma miR-655 concentration** | | | |  |
| --- | --- | --- | --- | --- | --- | --- |
| **Variables** | | **high** | **(n=48)** | **low** | **(n=56)** | ***P*-value^a^** |
| Gender | Female | 12 | (63%) | 7 | (37%) | 0.128 |
|  | Male | 36 | (42%) | 49 | (58%) |  |
| Age (60 years old) | ＜ 60 | 10 | (63%) | 6 | (37%) | 0.180 |
|  | 60 ≦ | 38 | (43%) | 50 | (57%) |  |
| T factor | T1,2 | 18 | (41%) | 26 | (59%) | 0.427 |
|  | T3,4 | 30 | (50%) | 30 | (50%) |  |
| N factor | N0 | 16 | (70%) | 7 | (30%) | **0.016** |
|  | N1,2,3 | 32 | (40%) | 49 | (60%) |  |
| Lymphatic invasion | ly0 | 23 | (62%) | 14 | (38%) | **0.023** |
|  | ly1,2,3 | 25 | (37%) | 42 | (63%) |  |
| Venous invasion | v0 | 20 | (57%) | 15 | (43%) | 0.145 |
|  | v1,2,3 | 28 | (41%) | 41 | (59%) |  |
| Tumor size | ＜50mm | 24 | (44%) | 31 | (56%) | 0.694 |
|  | 50mm ≦ | 24 | (49%) | 25 | (51%) |  |
| Histology | Well and moderately differentiated | 37 | (48%) | 40 | (52%) | 0.654 |
|  | poorly differentiated | 11 | (41%) | 16 | (59%) |  |
| Recurrences | Absent | 26 | (50%) | 26 | (50%) | 0.555 |
|  | Present | 22 | (42%) | 30 | (58%) |  |

**Additional file 10: Table S4.**

Association between plasma miR-655 levels and clinicopathological characteristics in ESCC patients who were pathologically classified into Stage II and III.

^a^ Chi-square or Fisher tests. NOTE: significant values are in bold.
