# Supplementary material for: Low levels of tumour suppressor miR-655 in plasma contribute to lymphatic progression and poor outcomes in oesophageal squamous cell carcinoma
Source: Mol Cancer. 2019 Jan 4;18:2. doi: 10.1186/s12943-018-0929-3 (PMC6320607; doi:10.1186/s12943-018-0929-3)
Supplement: Supplementary file 11 — Table S5. Association between lymphatic progression and clinicopathological characteristics in ESCC patients. (DOCX 19 kb) [file 12943_2018_929_MOESM11_ESM.docx]

|  | | **Lymphatic progression** | | | | **Univariate ^a^** |  | **Multivariate ^b^** | | |
| --- | --- | --- | --- | --- | --- | --- | --- | --- | --- | --- |
| **Variables** | | **Negative** | **(n=71)** | **Positive** | **(n=51)** | ***P*-value** |  | **OR ^c^** | **95%CI ^d^** | ***P*-value** |
| Gender | Female | 12 | (52%) | 11 | (48%) | 0.639 |  |  |  |  |
|  | Male | 59 | (60%) | 40 | (40%) |  |  |  | - |  |
| Age (60 years old) | ＜ 60 | 15 | (65%) | 8 | (35%) | 0.490 |  |  |  |  |
|  | 60 ≦ | 56 | (57%) | 43 | (43%) |  |  |  | - |  |
| T factor | T1,2 | 35 | (56%) | 27 | (44%) | 0.716 |  |  |  |  |
|  | T3,4 | 36 | (60%) | 24 | (40%) |  |  |  | - |  |
| Tumour size | ＜50 mm | 39 | (58%) | 28 | (42%) | 1.000 |  |  |  |  |
|  | 50 mm ≦ | 32 | (58%) | 23 | (42%) |  |  |  | - |  |
| Venous invasion | v0,1 | 51 | (55%) | 41 | (45%) | 0.296 |  |  |  |  |
|  | v2,3 | 20 | (67%) | 10 | (33%) |  |  |  | - |  |
| Histology | Well and moderately differentiated | 56 | (62%) | 35 | (38%) | 0.213 |  |  |  |  |
|  | Poorly differentiated | 15 | (48%) | 16 | (52%) |  |  |  | - |  |
| Plasma miR-655 level | High | 44 | (72%) | 17 | (28%) | **0.003** |  | 1 |  |  |
|  | Low | 27 | (44%) | 34 | (56%) |  |  | 3.25 | 1.55–7.05 | **0.001** |

**Additional file 11: Table S5.**

Association between lymphatic progression and clinicopathological characteristics in ESCC patients.

^a^ Univariate analysis was assessed using the Chi-square test and Fisher’s exact probability test. ^b^ Multivariate logistic regression was used to assess the risk factors for lymphatic progression. ^c^ OR: Odds ratio. ^d^ CI: confidence interval.

NOTE: significant values are in bold.
